# Voice Features of Sustained Phoneme as COVID-19 Biomarker

**DOI:** 10.1109/JTEHM.2022.3208057

**Published:** 2022-09-20

**Authors:** Nemuel D. Pah, Veronica Indrawati, Dinesh K. Kumar

**Affiliations:** Department of Electrical EngineeringUniversitas Surabaya 128202 Surabaya 60293 Indonesia; School of EngineeringRMIT University 5376 Melbourne VIC 3000 Australia

**Keywords:** COVID-19, voice features, sustained phoneme, support vector machine

## Abstract

Background: The COVID-19 pandemic has resulted in enormous costs to our society. Besides finding medicines to treat those infected by the virus, it is important to find effective and efficient strategies to prevent the spreading of the disease. One key factor to prevent transmission is to identify COVID-19 biomarkers that can be used to develop an efficient, accurate, noninvasive, and self-administered screening procedure. Several COVID-19 variants cause significant respiratory symptoms, and thus a voice signal may be a potential biomarker for COVID-19 infection. Aim: This study investigated the effectiveness of different phonemes and a range of voice features in differentiating people infected by COVID-19 with respiratory tract symptoms. Method: This cross-sectional, longitudinal study recorded six phonemes (i.e., /a/, /e/, /i/, /o/, /u/, and /m/) from 40 COVID-19 patients and 48 healthy subjects for 22 days. The signal features were obtained for the recordings, which were statistically analyzed and classified using Support Vector Machine (SVM). Results: The statistical analysis and SVM classification show that the voice features related to the vocal tract filtering (e.g., MFCC, VTL, and formants) and the stability of the respiratory muscles and lung volume (Intensity-SD) were the most sensitive to voice change due to COVID-19. The result also shows that the features extracted from the vowel /i/ during the first 3 days after admittance to the hospital were the most effective. The SVM classification accuracy with 18 ranked features extracted from /i/ was 93.5% (with F1 score of 94.3%). Conclusion: A measurable difference exists between the voices of people with COVID-19 and healthy people, and the phoneme /i/ shows the most pronounced difference. This supports the potential for using computerized voice analysis to detect the disease and consider it a biomarker.

Clinical and Translational Impact Statement: The outcomes of this research can aid in the development of an efficient screening device for COVID-19, as the testing is noninvasive and can be self-applied by patients using an application running on smartphones.

## I. Introduction

Covid-19 was declared a global pandemic by the World Health Organization (WHO) in March 2020 [1]. The pandemic rapidly spread to over more than 200 countries with more than 300 million confirmed cases and 5.5 million deaths by January 2022 [2]. The disease affects multiple body systems and organs [3], [4]. The main symptoms of COVID-19 are fever, dry cough, sore throat, dyspnea, fatigue, headache, and multiple organ failure in severe cases [4], [5].

The pandemic has caused enormous health, economic, and social challenges, and the effective suppression of its continued spread is dependent on efficient testing methods and strategies. The current gold standard for identifying infected people is based on molecular and serology testing. The polymerase chain reaction (PCR) test has been widely accepted as the most accurate COVID-19 test [6], [7], [8]. However, not all variants of the disease are serious, and variants such as Omicron are generally considered to have lower morbidity rates [9].

Since the seriousness of the disease is associated with the effect it has on the respiratory system [10], one of the methods used to detect severity is based on blood oxygen levels. However, this information is often too late. Thus, a need exists for inexpensive tools that can be used to detect COVID-19 in patients that present with respiratory system symptoms.

Although several methods for this purpose have been investigated as potential COVID-19 biomarkers [9], these are nonspecific and require complex or invasive procedures [10]. Likewise, several parameters have been investigated as potential COVID-19 biomarkers [11]. However, these are also nonspecific and require complex or invasive procedures. Alternatively, body temperature measurement has been widely practiced as a frontline screening method, but due to asymptomatic COVID-19 cases, it is insufficient as a primary means of COVID-19 screening or detection [12].

One promising biomarker for detecting COVID-19 in patients with a compromised respiratory system is voice signals. Voice has been proposed as a biomarker for diseases such as Parkinson’s disease, coronary artery disease, pulmonary hypertension, and chronic obstructive pulmonary disease [13], [14], [7]. The benefits of this method are that it is noninvasive, does not require physical contact with clinicians, and patients can easily record their voices without clinical assistance using a smartphone. Since COVID-19 affects the respiratory system, it creates distinct signatures in the patients’ voices [5]. COVID-19 patients may experience shortness of breath that results in the disruption of voice intensity [15]. The decrease in lung pressure changes the subglottal pressure that affects voice intensity and vocal fold vibration. Other COVID-19 symptoms, such as dry coughs and infection or inflammation in the oral or nasal cavity, may alter the vibration of the vocal folds as well as change the vocal tract frequency modulation. All the above changes will consequently modify the acoustic factors related to voice quality and, therefore, change the parameters in the patient’s voice.

Asiaee *et al.* [15] evaluated the change in voice quality of patients with COVID-19 using two-way ANOVA and Wilcoxon’s rank-sum test. They identified significant changes in cepstral peak prominence (CPP), harmonics measures (HNR and H1H2), the standard deviation of pitch, as well as time and amplitude perturbation. The parameters were evaluated on sustained vowel /a/ recorded from COVID-19 patients and healthy subjects of Persian speakers. Quatieri [16] found reduced complexity in the coordination of the voice production subsystem due to COVID-19. The author identified the change in speech envelope, CPP, pitch, and formant center frequency. These studies indicated the possibility of using voice parameters as COVID-19 biomarkers. However, the study by Asiaee *et al.* only focused on parameters related to vocal fold vibration of vowel /a/. Orlandic *et al.* [17] created a crowdsourcing dataset of over 25,000 cough recordings representing a wide range of COVID-19 statuses. This dataset provides the possibility for researchers to identify COVID-19 biomarkers.

Other researchers developed biomarkers using black-box approaches of deep learning. Suppakitjanusant [3] developed a deep-learning model to identify people infected with COVID-19 based on the log Mel spectrogram of vowel /a/, coughing, and polysyllabic words. The deep-learning classification with polysyllables achieved the best accuracy of 85%. Lower accuracies were produced if the model was given vowel /a/ of coughing parameters. A similar result was reported by Despotovic [18], who developed an ensemble and MLP model with 88.50% accuracy. Maor [7] constructed a CNN-based voice biomarker of COVID-19 using the Mel spectrogram. The biomarker achieved a classification AUC of 0.72. The above studies indicate the effectiveness of the voice Mel spectrum to indicate COVID-19. Verde [19] reported a machine learning that can classify COVID-19 patients with 97% accuracy, however, the study was based on unbalanced data. On the other hand, Loey [20] reported a deep-learning model that can classify COVID-19 patients with 95% accuracy based on the parameters of coughing.

As the research in this area is still in the preliminary stage, more studies are needed to identify a reliable COVID-19 biomarker extracted from voice features that could be implemented as operable devices or testing procedures. The above research indicates a possible biomarker in the voice parameter. However, the studies investigated some limited voice features and extracted only from vowel /a/. Furthermore, the use of voice features in COVID-19 identification may lead to over-optimistic or misleading results due to demographic, subjective, and acoustic bias as shown in the work of Han *et al.* [21]. To limit the bias this study extracted voice parameters from only sustained phonemes.

Expanding on previous findings, this study investigated a wider range of features related to voice production mechanisms or organs, including the features related to air pressure production by the lung, vocal cord vibration, and voice modulation in the vocal tract (oral and nasal cavity). This study also extracted the features from a wider range of sustained phonemes to capture any possible alteration due to COVID-19 that might occur in voice production mechanisms and organs.

This study aimed to determine the most effective features that could be used as a COVID-19 biomarker. Once these features are identified, they can be used to develop a noninvasive device or testing procedure to screen people infected with COVID-19.

## II. Materials and Methods

### A. Participants

The sustained phonemes were recorded from 40 (21 males and 19 females) COVID-19 patients (CV) and 48 (21 males and 27 females) age-matched healthy participants (HC) as the control group. The CV patients were hospitalized in the COVID-19 ward of Husada Utama Hospital in Surabaya, Indonesia in June and July 2021. The period was the beginning of the second wave of the COVID-19 pandemic in Indonesia, which was dominated by the Delta variant [22]. Each CV patient was confirmed with a positive result of the reverse-transcriptase polymerase chain reaction (RT-PCR) test performed upon admission by the hospital.

The CV patients tested positive and had one or more symptoms of COVID-19 (e.g., fever, cough, sore throat, malaise, headache, muscle pain, nausea, vomiting, diarrhea, loss of taste and smell). About 52% (21 patients) of the CV group were given 3–5 LPM of oxygen supplementation due to shortness of breath with SpO_2_ ≥ 94%. Seven CV patients were given 8 LPM of oxygen supplementation with SpO**2** of less than 94%, while the other CV patients did not need oxygen supplementation. All 40 patients had recovered from COVID-19 following hospitalization.

The HC participants were recruited randomly from people who had never been diagnosed with COVID-19, had no history of any disease related to respiration or voice production mechanism, and did not have any COVID-19 symptoms within 14 days before and after the recording.

The study protocol complied with the Helsinki Declaration and was approved by the Institutional Ethics Committee of the University of Surabaya, Surabaya, Indonesia (159/KE/V/2021) and Husada Utama Hospital, Surabaya, Indonesia (582/RSHU/Dir./V/2021). Before the experiments, written informed consent was obtained from all the participants. Table 1 presents participants’ demographic and clinical information.

**TABLE 1:** Participants’ Demographics

	CV		HC		ANOVA p-value
	Male	Female	Male	Female	
# Participants	21	19	21	27	
Age (years)	44.5 ± 18.7	42.9 ± 13.7	43.7 ± 15.2	45.9 ± 12.8	0.921

### B. Phoneme Recording

Six sustained phonemes (i.e., /a/, /e/, /i/, /o/, /u/, and /m/) were recorded from each participant. These phonemes were selected to examine a wide range of voice production aspects, including the nasal voice. All the participants were asked to produce the phonemes as long as it was comfortable within a single breath at their natural pitch and loudness while keeping the tone as flat as possible.

The phonemes of CV patients were recorded by two nurses from the hospital who were trained for the data collection using an Android application developed in this study. The application recorded the phonemes via the phone’s microphone and the recordings were saved in a single-channel 3GP format with a sampling rate of 8 kHz and a 32-bit sampling quantization. The sampling rate was selected to support the aim of this study, which is to develop a system that would be functional with minimum resources, such that these can also be used in less affluent societies. The 8 kHz sampling is the norm for 2G/3G phones and hence was chosen for this study. The files were transferred to the FireBase cloud database. The duration of each recording was between 3 to 15 seconds. The recording was performed in COVID-19 hospital wards while keeping the ambient noise as low as possible. The average SNR of the recordings was 27.80 dB.

The six sustained phonemes were expected to be recorded from the CV patients once every day while hospitalized. However, due to the patients’ health conditions and some technical considerations, the recording could not be properly acquired from each CV patient every day as expected. Table 2 provides the list of valid phoneme recordings from each CV patient during their stay in the hospital.

**TABLE 2:** Falid Phoneme Recordings From CV Patients

ID	Gendei		O2 Supl eme nlation	Day 1	Day 2	Day 3	Day 4	Day 5	Day 6	Day 7	Day 8	Day 9	Day ID	Day 11	Day 12	Day 13	Day 14	Day 15	Day 16	Day 17	Day 18	Day 19	Day 20	Day 21	Day 22
1	M	66	-													aeiou-									
2	F	46	-								aeioum		aeioum												
3	F	38	-			aeiou-			aei-um		aeioum														
4	F	63	-					aei-u-																	
5	M	13	-										aeioum			ae-num									
6	F	16	-										aeiou-			aeioum									
7	M	40	8 Ipm							-eioum															
8	M	65	8 Ipm									-eioum													
9	F	43	8 Ipm										aeioum		aeioum		aeioum	aeioum		aeioum					
10	M	67	8 Ipm										a-i-u-		aeio-m	-eioum	aeioum	aeioum	aeioum						
11	M	50	8 Ipm												aeiou-		aeioum		aeioum	aeioum					
12	F	23	-	aeiou-					–i—																
13	M	19	3-5 Ipm	aeinu-	-eioum	aeioum	aeioum	aeioum	aeioum			aeioum	aeioum							aeioum	aeioum		aeioum		
14	M	78	3-5 Ipm	ae-o-m	ae-o-m	ae-o-m	ae-o-m	ae-o-m	ae-o-m			ae-o-m								ae-o-m	ae-oum		ae-oum		
15	F	58	8 Ipm	ae-os–;	ae-ou-	aeioum		aeioum			a-iou-	-eioum						aeioum		-eioum		aeioum			
16	M	57	3-5 Ipm	aeiou-	aeioum	aeioum					aeiou-	-eio-m					aeioum	aeioum							aeioum
17	M	39	8 Ipm		aeiou-	aeioum				aeiou-	aeioum						aeiou-	aeioum		aeioum					
18	F	55	3-5 Ipm	aeioum	aeioum	aeioum		aeioum	aeioum	aeioum		aeioum				aeioum	aeioum								
19	F	35	3-5 Ipm	aeioum	aeioum	aeioum		aeioum	aeioum	aeioum		aeioum				aeioum									
20	M	40	3-5 Ipm	aeioum		aeioum		aei-um																	
21	M	27	3-5 Ipm	aeioum	aeioum	aeioum	aeioum	aeioum	aeioum	aeioum	aeioum	aeioum													
22	F	53	3-5 |pm	aeioum	aei-um	ae-o-m			aeioum	aeioum		aeioum				aeioum		aeioum					aeioum		
23	M	51	3-5 Ipm	aeioum	aeiou-	aeioum		aeioum	-eioum	aeio-m		aeioum				-ei-um	aeioum	aeioum							
24	F	65	3-5 Ipm	aeioum		aeioum		aeiou-	-eioum	aeioum							aeioum							aeioum	
25	M	15	3-5 Ipm	aeioum	aeioum		aeioum	aeioum	aeioum		aeioum														
26	M	50	3-5 Ipm	aeioum	aeioum		aeioum	aeioum	-eioum		aeioum														
27	M	38	3-5 Ipm	aeiou-	aeioum		aeioum	aeioum	aeioum						aeioum										
28	F	33	3-5 Ipm	aeioum			aeioum	-eioum	-eioum						aeioum	aeioum									
29	M	57	-	aeioum		aei-um	aeioum																		
30	F	37	-	aeiou-		aeiou-	aei-u-	aeiou-						aeiou-	aeiou-										
31	F	46	3-5 Ipm	a-i-u-		aeioum	aeioum	aeioum		aeioum					aeioum	aeioum									
32	M	17	-	aeioum		aeiou-	aeioum	aeioum		aeiou-					aeiou-	aeioum									
33	F	57	3-5 Ipm	aeiou-	aeiou-	-eioum						aeioum													
34	F	37	3-5 Ipm	aeioum	aeioum	aeiou-						aeioum	aeioum		aeioum										
35	M	28	3-5 Ipm	aeiou-	aeiou-																				
36	M	59	-	–io–	aeiou-		aeioum											aeiou-							
37	M	59	-	aeiou-									aeioum					aeiou-							
38	F	55	3-5 Ipm	-eioum							aeioum														
39	F	28	3-5 |pm	-eioum						aeioum	aeioum	-eioum													
40	F	27	3-5 Ipm	aeiou-	aeiou-				aei-um																

The recording of HC participants was acquired using the same Android application with a similar setting of 8 kHz and 32-bit resolution. The recording process occurred in a common room while the ambient noise was kept at the lowest possible level (mean SNR = 30.10 dB).

### C. Features Extraction

Before the feature extraction process, each recording was manually observed using Audacity, an open-source sound editing software. A segment of 1.0 seconds with a clean phoneme recording was extracted from each segment. The uniform duration of 1.0 seconds was selected based on the optimum length of recording without interference from other sounds in the hospital ward. The 1.0 seconds segment of each recording was converted to WAV format at a sampling rate of 8 kHz and 32-bit resolution.

A Praat [23] code was used to extract all voice features from the recordings. The extraction process was performed using the Praat default settings with a pitch range from 75 to 600 Hz. Thirty-four features were extracted from each recording as shown in Table 3. Jitter [24], Shimmer [24], SD of pitch frequency, and the harmonics features were expected to capture the change in vocal cord vibration due to COVID-19 infection. The features correspond to the time and frequency perturbation, and noise of glottal vibration [24].

**TABLE 3:** List of Voice Features Extracted From the Recordings

No	Feature (unit)	Description
1	Jitter-abs (s)	Absolute time perturbation of glottal pulses
2	Jitter-rel (%)	Relative time perturbation of glottal pulses
3	Shimmer-abs (dB)	Absolute amplitude perturbation of glottal pulses
4	Shimmer-rel (%)	Relative amplitude perturbation of glottal pulses
5	Pitch-SD (Hz)	SD of pitch frequency
6	HNR (dB)	Harmonics-to-noise ratio
7	NHR	Noise-to-harmonics ratio
8	F1-mean (Hz)	Mean of formants F1 frequency
9	F2-mean (Hz)	Mean of formants F2 frequency
10	F3-mean (Hz)	Mean of formants F3 frequency
11	F4-mean (Hz)	Mean of formants F4 frequency
12	F1-SD (Hz)	SD of formants F1 frequency
13	F2-SD (Hz)	SD of formants F2 frequency
14	F3-SD (Hz)	SD of formants F3 frequency
15	F4-SD (Hz)	SD of formants F4 frequency
16	VTL-F1 (cm)	Apparent vocal tract length based on F1
17	VTL-F2 (cm)	Apparent vocal tract length based on F2
18	VTL-F3 (cm)	Apparent vocal tract length based on F3
19	VTL-F4 (cm)	Apparent vocal tract length based on F4
20	Intensity-mean (dB)	Mean of voice intensity
21	Intensity-SD (dB)	SD of voice intensity
22–34	MFCC (13 coefficients)	Mell Frequency Cepstral Coefficients

The formants features (F1 to F4) [25], the apparent vocal tract length [26], [27], and the 13 coefficients of MFCCs [28] represent the change in vocal tract formation due to COVID-19. The voice intensity is controlled by the subglottal pressure, which is controlled by the respiratory muscles and lung volume [29], and thus the intensity features were expected to represent a change in lung condition due to COVID-19.

### D. Statistical Analysis

The effectiveness of the features to separate CV from HC subjects was firstly assessed using statistical analysis. The statistical analyses were performed using MATLAB 2018b (MathWorks). The normality of the extracted features was examined with the Anderson-Darling test [30]. Mann-Whitney U test [31] was used to compare the group differences for each of the features and phonemes between the CV and HC groups. The 95% confidence level was considered for the analysis and a p-value < 0.05 indicated that the mean of the groups was significantly different. The differences between the groups were also examined using effect size (ES) [32]. The ES between two groups of data (A and B) was calculated using Cohen’s $d$ [33] in eq. (1).

\begin{equation*} d=\frac {\bar {X}_{A}-\bar {X}_{B}}{\sqrt {\frac {\left ({n_{A}-1 }\right){SD}_{A}{}^{2}+\left ({n_{B}-1 }\right){SD}_{B}{}^{2}}{{(n_{A}+ n_{B}-2)}}}}\tag{1}\end{equation*}

An ES of 0.50 or above indicates a medium to a large difference between the compared groups.

### E. Classification Method

The effectiveness of the voice features to separate CV from HC subjects was also be examined based on the feature’s performance in a Support Vector Machine (SVM) [34] classifier. The SVM used in this work was trained with a Gaussian kernel and validated using “leave-one-subject-out” (LOSO) cross-validation. The Gaussian kernel was selected because it showed the best result compared to the other kernels.

Several combinations of voice features were selected to be used in the SVM training and validation. The accuracy, sensitivity, and selectivity were recorded as the measure of the features’ effectiveness as a COVID-19 biomarker. The feature selection was based on the statistical analysis and a rank calculated by ReliefF algorithm [35]. The ReliefF algorithm ranks the features based on $k$ nearest hits and misses and averages their contribution to the weights of each feature. The ReliefF algorithm was implemented using MATLAB 2018b with 10 nearest neighbors ( $k = 10$).

## III. Results

### A. Statistical Analysis

The result of the Anderson-Darling normality test showed that most of the features were not normally distributed, and thus the Mann-Whitney U test, a nonparametric test, was used to test for group differences in each of the features. The group differences were also examined by calculating the ES. In this analysis, a feature is considered significant if the Mann-Whitney U test p-value was equal to or less than 0.05 and the ES was 0.50 or above. Table 4 provides the ES and the results of the Mann-Whitney U test between CV and HC for all the features. The numbers in the table are the ES of the significant features (features with p-value ≤ 0.05 and ES ≥ 0.50).

**TABLE 4:** The Effect Size and Mann Whitney U-Test Results of Voice Features

Features	Phoneme (All recordings)						Phoneme (Recorded on Days 1, 2, and 3)						Phoneme (Recorded on Days 4, 5, and 6)					
	/a/	/e/	/i/	/o/	/u/	/m/	/a/	/e/	/i/	/o/	/u/	/m/	/a/	/e/	/i/	/o/	/u/	/m/
Jitter-abs	-	-	-	-	-	-	0.52	-	-	-	-	-	-	-	-	-	-	-
Jitter-rel	-	-	-	-	-	-	-	-	-	-	-	-	-	-	−0.63	-	-	-
Shimmer-abs	-	-	-	-	−0.53	-	-	-	-	-	−0.53	-	-	−0.70	−0.73	−0.80	−1.09	-
Shimmer-rel	-	-	-	-	−0.50	-	-	-	-	-	−0.51	-	-	−0.71	−0.74	−0.78	−0.99	-
Pitch-SD	-	-	-	-	-	-	-	-	-	-	-	-	-	-	-	-	-	-
HNR	−0.56	-	-	-	-	-	−0.75	-	-	-	-	-	-	-	-	0.60	0.91	-
NHR	-	-	-	-	-	-	-	-	-	-	-	-	-	-	-	−0.52	-	-
F1-mean	-	-	−0.56	-	-	-	-	−0.60	−0.89	-	−0.67	−0.73	-	-	-	-	-	−0.54
F2-mean	0.51	-	-	-	-	-	-	-	-	-	-	-	1.29	-	-	0.77	0.63	0.70
F3-mean	-	-	-	-	-	-	-	-	-	-	-	-	-	-	-	-	−0.80	-
F4-mean	-	-	-	-	-	-	0.55	-	-	0.56	-	-	-	-	−0.71	-	−1.06	-
F1-SD	-	−0.54	−0.60	-	-	-	0.64	−0.73	−0.61	-	-	-	-	−1.00	−1.03	−0.61	−0.69	-
F2-SD	-	-	−0.66	-	-	-	-	-	−0.62	-	-	-	-	-	−1.14	-	-	−0.71
F3-SD	0.50	−0.55	−0.56	-	-	-	0.71	−0.78	-	-	-	-	-	−0.82	−1.16	-	-	-
F4-SD	-	-	−0.80	-	-	-	-	-	−0.84	-	-	-	-	-	−1.34	-	-	-
VTL-F1	-	-	0.58	-	-	-	-	0.65	0.86	0.52	0.66	0.84	-	-	-	-	-	0.55
VTL-F2	−0.61	-	-	-	-	-	-	-	-	-	-	-	−1.39	-	-	−0.82	−0.59	−0.69
VTL-F3	-	-	-	-	-	-	-	-	-	-	-	-	-	-	-	-	0.77	-
VTL-F4	-	-	-	-	-	-	−0.57	-	-	−0.56	-	-	-	-	0.68	-	1.06	-
Intensity-mean	-	-	-	-	-	-	-	-	-	-	-	0.83	-	-	-	-	0.86	0.86
Intensity-SD	-	-	-	-	−0.56	−0.62	-	-	-	-	−0.68	−0.91	-	−0.67	−0.88	−0.79	−1.39	−1.24
MFCC-c0	0.55	0.74	0.57	-	-	-	0.72	0.72	-	-	-	-	0.97	1.42	1.33	0.70	0.96	0.79
MFCC-c1	-	-	-	-	-	-	-	-	-	-	-	-	−0.65	−0.79	−0.85	-	−0.51	−0.55
MFCC-c2	-	-	-	-	-	-	-	-	-	-	-	-	−0.54	-	-	−0.57	−0.66	−0.66
MFCC-c3	-	-	0.52	-	0.53	0.56	-	-	-	-	0.57	-	0.75	0.63	0.68	-	0.73	0.80
MFCC-c4	1.15	0.87	1.07	0.60	-	0.58	1.50	1.15	1.49	0.64	-	0.93	1.51	0.88	0.89	0.65	-	0.74
MFCC-c5	-	-	-	-	-	-	−0.62	−0.67	-	-	-	-	−0.86	-	-	-	-	-
MFCC-c6	-	-	-	-	-	-	−0.70	-	-	-	-	-	−0.51	−0.68	-	-	-	-
MFCC-c7	-	-	-	-	-	-	-	-	-	−0.52	-	-	-	-	-	-	0.59	-
MFCC-c8	-	-	-	-	-	-	-	-	-	-	-	-	-	-	-	-	-	-
MFCC-c9	-	-	-	-	-	-	-	-	-	-	-	-	-	-	-	-	-	-
MFCC-c10	-	-	-	-	-	-	-	-	-	-	-	-	-	-	-	-	-	-
MFCC-c11	-	-	-	-	-	-	-	-	-	-	-	-	-	-	-	-	-	-
MFCC-c12	-	-	-	-	-	-	-	0.51	-	-	-	0.55	-	-	-	-	-	-

Note: The number indicates the features with Mann-Whitney U test p-value ≤ 0.05 and ES ≥ 0.50, while ‘−‘ indicates features with p-value > 0.05 or ES < 0.50.

The table presents the significant features when analyzed using all the recordings (Days 1–22) and recordings from Days 1–3 only and Days 4–6 only. The table shows that the voice features were less sensitive to the COVID-19 biomarker if all the recordings were included in the analysis. Only 27 significant features were found with an average |ES| of 0.63. The number of significant features was increased to 41 (average |ES| = 0.72) when the statistical analysis only considered the features extracted from the phoneme recorded on the first 3 days after testing positive with COVID-19. The highest number of significant features was observed on the phoneme recorded on Days 4–6 after testing positive (73 significant features, average |ES| = 0.83).

**FIGURE 1.**
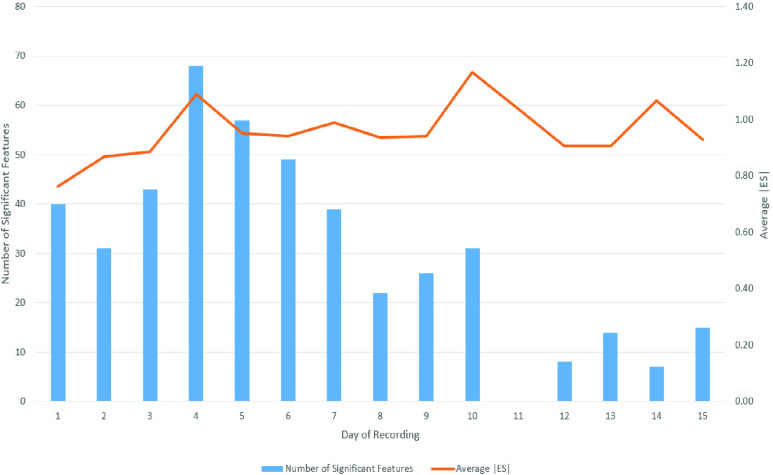
The number of significant features and average effect size (ES) of statistical test between control subjects (HC) and COVID-19 patients (CV) for each day of recordings.

Figure 1 presents the number of significant features and average |ES| for each day of recordings. The recordings from Day 4 contain the most significant features to discriminate CV patients from HC participants. Features from Days 4–6 recordings were the most effective features to mark COVID-19. The phonemes recorded after Day 7 were not effective to identify COVID-19.

**FIGURE 2.**
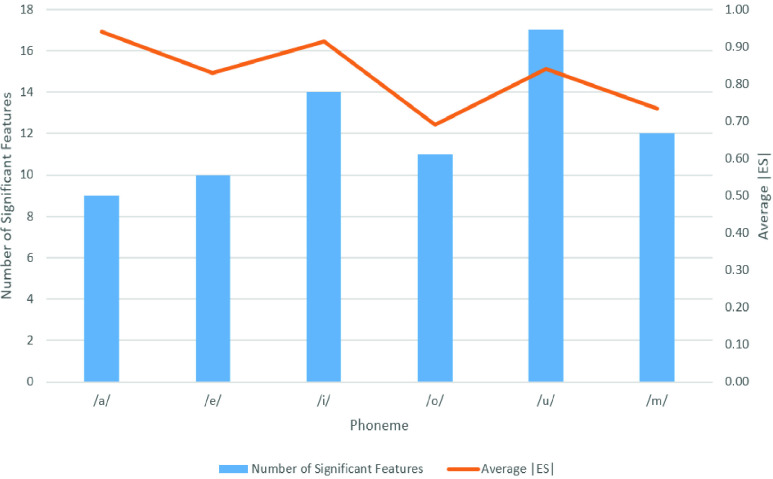
The number of significant features and average effect size (ES) of statistical test between control subjects (HC) and COVID-19 patients (CV) for each phonemes recorded on days 4 to 6.

Figure 2 compares the effectiveness of each phoneme in differentiating CV and HC based on the recordings on Days 4–6. The figure shows that the significance of the six phonemes was relatively equal. Phoneme /a/ had the highest average |ES| but with the least number of significant features. On the other hand, phoneme /u/ had the highest number of significant features but with a low average |ES|. Phoneme /i/ was the most effective phoneme with a relatively high number of significant features with a relatively high average |ES|.

Table 4 shows that the features corresponding to frequency modulation of vocal tracts (MFCC c0 to c4, formants, and VTL) were more sensitive to a change in voice due to COVID-19. The amplitude perturbation (shimmer) and STD of voice intensity were also significantly affected by COVID-19.

### B. SVM Classification

**FIGURE 3.**
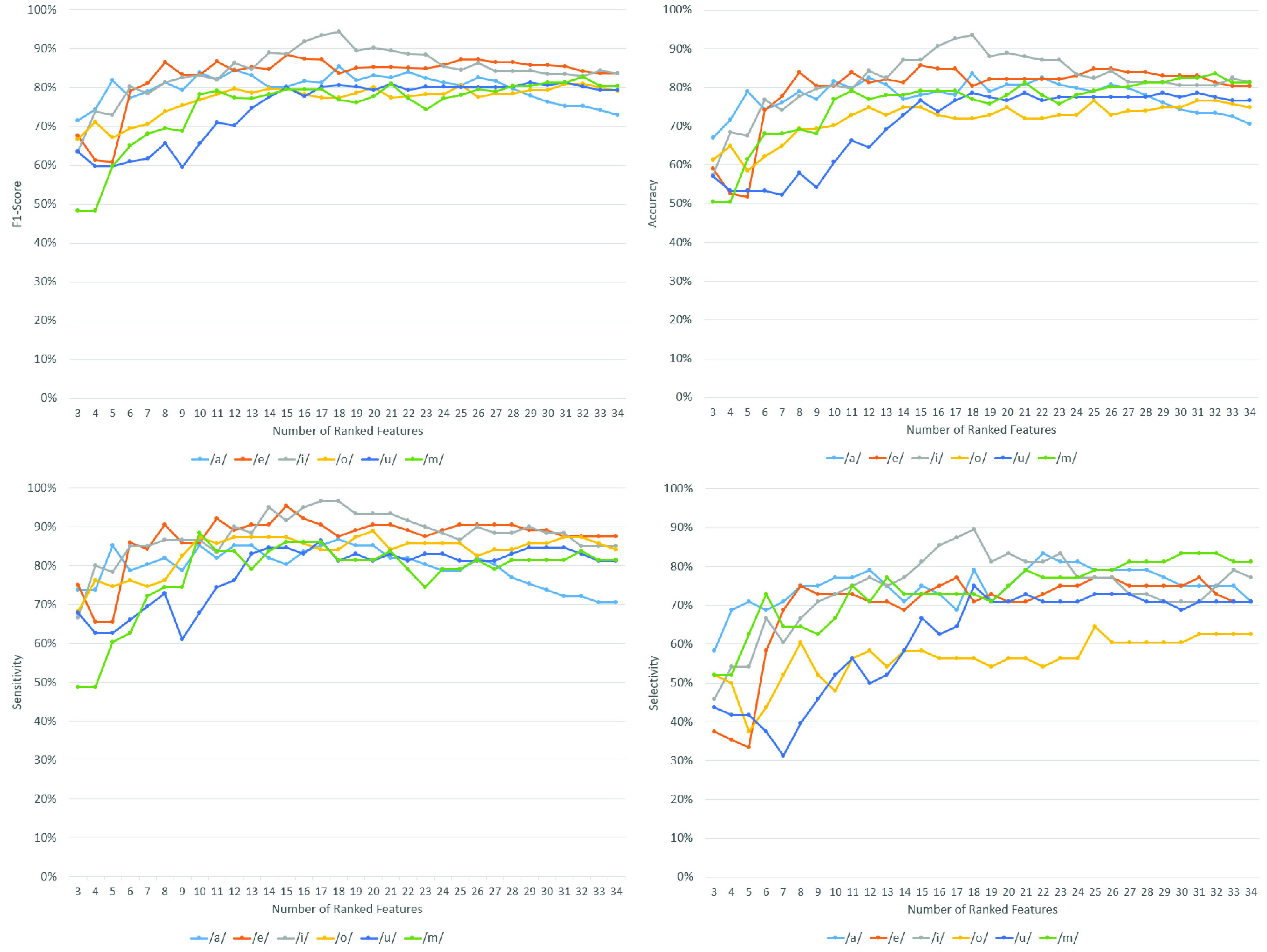
The performance (F1 score, accuracy, sensitivity, and selectivity) of SVM classification with different number of ranked features. The input to SVM is the recordings from Days 1–3.

Figure 3 presents the performance of SVM classification (F1-score, accuracy, sensitivity, and selectivity) with a different number of ranked features as the inputs. The features were ranked using the ReliefF algorithm with $k = 10$ nearest neighbors. The ranked features for the six phonemes are shown in Table 5. The features were extracted from the phonemes recorded from the CV patients in the first 3 days after being admitted to the hospital. The SVM classification of these recordings outperformed the classification results if using the recordings from Days 4–6 or if the whole recordings (Days 1–22) were considered.

**TABLE 5:** The Ranked Features of Days 1–3 Recordings Calculated Using ReliefF

Rank	/a/	/e/	/i/	/o/	/u/	/m/
1	VTL-F4	MFCC-c4	MFCC-c4	VTL-F4	MFCC-c1	Intensity-SD
2	F4-mean	VTL-F4	VTL-F4	MFCC-c3	F4-SD	F1-SD
3	MFCC-c4	F4-mean	F4-mean	F4-mean	MFCC-c9	VTL-F2
4	MFCC-c3	Intensity-mean	F2-mean	MFCC-c11	F2-mean	F2-mean
5	MFCC-c11	Pitch-SD	VTL-F2	MFCC-c1	VTL-F2	MFCC-c1
6	F2-mean	F2-SD	Intensity-SD	F2-mean	VTL-F3	F3-mean
7	F3-SD	MFCC-c0	MFCC-c0	VTL-F2	F3-mean	MFCC-c4
8	F1-mean	Intensity-SD	F4-SD	MFCC-c2	MFCC-c3	F4-SD
9	F2-SD	MFCC-c3	VTL-F3	VTL-F3	MFCC-c0	F2-SD
10	MFCC-c6	MFCC-c2	F3-mean	F2-SD	F3-SD	VTL-F4
11	F4-SD	F4-SD	Jitter-abs	F3-SD	F1-mean	MFCC-c11
12	VTL-F3	VTL-F1	MFCC-c10	MFCC-c0	MFCC-c7	F4-mean
13	F3-mean	F1-mean	MFCC-c6	F4-SD	MFCC-c2	VTL-F3
14	MFCC-c7	MFCC-c1	MFCC-c3	Jitter-abs	VTL-F1	MFCC-c3
15	F1-SD	F3-SD	Jitter-rel	F3-mean	VTL-F4	F1-mean
16	MFCC-c2	F3-mean	MFCC-c5	MFCC-c10	MFCC-c4	Jitter-rel
17	MFCC-c1	VTL-F3	MFCC-c1	MFCC-c12	F4-mean	F3-SD
18	VTL-F1	MFCC-c5	Pitch-SD	HNR	Intensity-SD	NHR
19	MFCC-c12	MFCC-c12	F1-mean	Pitch-SD	MFCC-c10	Jitter-abs
20	MFCC-c0	HNR	F3-SD	MFCC-c4	F1-SD	MFCC-c5
21	Intensity-mean	VTL-F2	F2-SD	MFCC-c6	MFCC-c8	Pitch-SD
22	Jitter-abs	F2-mean	F1-SD	Intensity-mean	F2-SD	MFCC-c12
23	HNR	MFCC-c6	MFCC-c2	MFCC-c9	HNR	VTL-F1
24	VTL-F2	MFCC-c9	Intensity-mean	MFCC-c5	Jitter-abs	MFCC-c2
25	NHR	MFCC-c11	MFCC-c11	MFCC-c7	MFCC-c11	Shimmer-rel
26	Jitter-rel	MFCC-c10	VTL-F1	F1-mean	Pitch-SD	Shimmer-abs
27	MFCC-c10	NHR	Shimmer-abs	VTL-F1	NHR	MFCC-c10
28	Pitch-SD	Shimmer-rel	Shimmer-rel	Intensity-SD	Intensity-mean	Intensity-mean
29	MFCC-c5	Shimmer-abs	MFCC-c8	Shimmer-abs	Jitter-rel	MFCC-c0
30	Intensity-SD	F1-SD	MFCC-c7	Jitter-rel	MFCC-c5	HNR
31	MFCC-c8	MFCC-c7	NHR	Shimmer-rel	Shimmer-abs	MFCC-c9
32	Shimmer-abs	Jitter-rel	HNR	NHR	MFCC-c12	MFCC-c8
33	Shimmer-rel	Jitter-abs	MFCC-c9	MFCC-c8	Shimmer-rel	MFCC-c6
34	MFCC-c9	MFCC-c8	MFCC-c12	F1-SD	MFCC-c6	MFCC-c7

**FIGURE 4.**
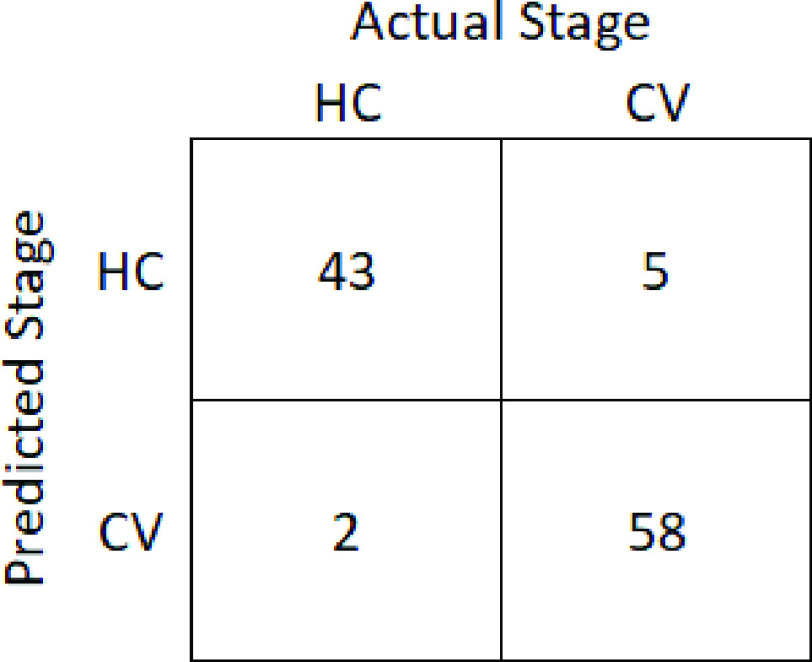
Confusion matrix of SVM classification between COVID-19 patients (CV) and control subjects (HC) based on 18 ranked features of phoneme /i/ recorded during the first 3 days in the hospital.

The figure shows that SVM with input features extracted from phoneme /i/ produced the highest classification performance. F1 scores of more than 90% were achieved with 16 to 21 ranked features. The highest SVM classification performance was achieved with 18 ranked features of /i/ (F1 score = 94.3%, accuracy = 93.5%, sensitivity = 96.7%, selectivity = 89.6%). Figure 4 provides the confusion matrix of the classification.

The 18 ranked features of /i/ are shown in the fourth column of Table 5 and indicate the highest-ranked features were dominated by features related to vocal tract filtering (e.g., MFCC, VTL, and formants) and the stability of the respiratory muscles and lung volume (Intensity-SD). Among the MFCC features, the MFCC-c4 was the most effective feature. This result suggests that features related to vocal tract modulation carry the most information as COVID-19 biomarkers. This result was consistent with the statistical analysis.

## IV. Discussion

Several studies had reported the possibility of using voice features as COVID-19 biomarkers [3], [7], [15], [16], [18]. This study investigated a range of voice features that were related to vocal cord vibration (jitter, shimmer, SD of pitch, HNR, and NHR), vocal tract modulation (formants, VTL, and MFCC), and lung function (intensity). In this work, the authors extracted the features from six sustained phonemes (i.e., /a/, /e/, /i/, /o/, /u/, and /m/). These phonemes were selected to examine the whole aspect of the voice production system.

The statistical analysis and SVM classification indicated that the voice features of sustained phoneme corresponding to vocal tract modulation (MFCC, formants, and VTL) and lung pressure stability (Intesity-SD) were sensitive to COVID-19 infection and, therefore, could potentially be adopted as a COVID-19 biomarker compared to the features of vocal fold vibration (jitter, shimmer, pitch, HNR, and NHR). The results suggest that COVID-19 symptoms that affect laryngeal activity and the oral and nasal cavities create the most alteration to the voice quality of sustained phonemes. This result explained the findings of Suppakitjanusant [3], Quatieri [16], Maor [7], and Loey [20] that parameters related to frequency modulation of the vocal tract (log Mel spectrogram, formants, and scalogram) contributed significantly to the performance of the classifiers. The low to medium MFCC coefficients (c0, c3, c4, c5, c6, and c10) were the most sensitive features. These coefficients represent vocal tract impulse responses in the range of low to medium frequency [36].

Among the investigated phonemes, the features extracted from /i/ were the most effective features to distinguish COVID-19 patients from healthy subjects. A large number of features from /i/ produced a p-value of less than 0.05 and a relatively high average |ES|. The SVM classification with features extracted from /i/ produced the highest F1 score of 94.3%.

The phoneme /i/ is a cardinal vowel produced while the tongue is at a high-front position with spread lips [37], [38]. The tongue is very close to the hard palate while its sides are pressed against the teeth. The production of /i/ requires precise control of the air gap between the tongue and hard palate as well as maintaining proper lips position and shape. In contrast, the vowel /a/, which was used commonly in the previous studies, is a back-open cardinal vowel that requires less precise control as long as the jaw is open wide and the tongue is at the lowest position. Any change of vocal tract muscle control due to infection, pain, or inflammation caused by COVID-19 will, therefore, affect the production of /i/ more than /a/.

The statistical analysis of features extracted from the phonemes recorded on Days 4–6 shows better separation between COVID-19 patients and healthy subjects, followed by the recordings from Days 1–3. On the other hand, SVM classification gave the best classification with recordings from Days 1–3. The difference between these two approaches was because statistical analysis attempted a linear separation, whereas SVM with Gaussian kernel used a nonlinear approach. These results suggest that the most sensitive COVID-19 biomarkers were possibly extracted from voice recordings during the first 6 days after testing positive. This result agrees with the COVID-19 life-cycle and symptoms [39].

The novelty of this study is the finding that sustained phoneme features related to frequency modulation in the vocal tract contains the most information to be used as COVID-19 biomarkers. The other significant novelty is that the features extracted from /i/ gave better differentiation between COVID-19 patients and healthy subjects. This study also indicates that the features recorded in the first 6 days gave the best results.

The limitation of this study is that this study investigated a relatively small number of subjects in the hospital environment. Due to the condition of the patients, the recordings could not be taken every day from all the patients. Further study needs to be conducted with a large number of patients under a standardized recording environment and protocol. The other limitation of this study is that the recordings were taken after the patients tested positive with RT-PCR. It could be more useful if the recordings were taken from the subjects before being declared COVID-19 positive by other means. Therefore, the features will be reliable as COVID-19 screening parameters.

## V. Conclusion

This study investigated the effectiveness of features extracted from six sustained phonemes to differentiate people infected with COVID-19 from healthy subjects. The findings indicate the most effective features were those related to vocal tract modulation from sustained phoneme /i/. The highest SVM classification accuracy (93.5%) was achieved with 18 ranked features extracted from phoneme /i/ recorded during the first 3 days after being admitted to the hospital. The results from this study have the potential for developing a noninvasive device or testing procedure that can be developed to screen people infected with COVID-19.
